# Exploring QRS Area beyond Patient Selection in CRT—Can It Guide Left Ventricular Lead Placement?

**DOI:** 10.3390/jcdd11010018

**Published:** 2024-01-11

**Authors:** Frederieke Eerenberg, Justin Luermans, Joost Lumens, Uyên Châu Nguyên, Kevin Vernooy, Antonius van Stipdonk

**Affiliations:** 1 Department of Cardiology, Cardiovascular Research Institute Maastricht (CARIM), Maastricht University Medical Center+, 6229 ER Maastricht, The Netherlands; justin.luermans@mumc.nl (J.L.); u.nguyen@maastrichtuniversity.nl (U.C.N.); kevin.vernooy@mumc.nl (K.V.); twan.van.stipdonk@mumc.nl (A.v.S.); 2 Cardiovascular Research Institute Maastricht (CARIM), University Maastricht (UM), 6229 ER Maastricht, The Netherlands; joost.lumens@maastrichtuniversity.nl

**Keywords:** vectorcardiogram (VCG), QRS area, cardiac resynchronization therapy (CRT), left ventricular lead placement (LVLP)

## Abstract

Vectorcardiographic QRS area is a promising tool for patient selection and implantation guidance in cardiac resynchronization therapy (CRT). Research has mainly focused on the role of QRS area in patient selection for CRT. Recently, QRS area has been proposed as a tool to guide left ventricular lead placement in CRT. Theoretically, vector-based electrical information of ventricular fusion pacing, calculated from the basic 12-lead ECG, can give real-time insight into the extent of resynchronization at any LV lead position, as well as any selected electrode on the LV lead. The objective of this review is to provide an overview of the background of vectorcardiographic QRS area and its potential in optimizing LV lead location in order to optimize the benefits of CRT.

## 1. Introduction

Electrical conduction disturbances can lead to dyssynchronous contractions of the heart and ultimately lead to heart failure (HF). Heart failure is a worldwide problem that is exposing itself on an expanding scale. The number of patients with HF has increased by approximately 23% over the past 10 years [1,2,3,4]. Dyssynchronous HF diagnosis originates from a delayed activation of the left ventricular free wall relative to the intraventricular septum, leading to a dyscoordinated contraction and hence reduced left ventricular ejection fraction (LVEF) [5]. Through stimulation of the left ventricle (LV) and right ventricle (RV) (i.e., biventricular pacing), cardiac resynchronization therapy (CRT) aims to overcome the conduction disturbances causing the dyssynchrony. CRT has proven to improve morbidity, mortality and quality of life in the long term [6].

Current, guidelines state that symptomatic HF patients who have less than, or equal to 35% left ventricular ejection fraction (LVEF), despite optimal medical therapy (OMT) and signs of dyssynchrony based on QRS duration and QRS morphology, may be considered for CRT. The use of QRS duration and QRS morphology derived from a 12-lead ECG contains difficulties with respect to the interpretation and specification of the vector direction of the conduction delay. QRS area, a vectorcardiographic biomarker, which provides insight into ventricular conduction delay, which can be reconstructed based on the 12-lead ECG, has been proposed as a superior marker of true LV dyssynchrony. Several observational studies have shown that QRS area is superior compared to QRS duration and QRS morphology in providing insight into the benefit derived from CRT [7,8]. However, for deployment in clinical practice, further study is required. The primary factors that have been recognized to contribute to the extent of the benefit of CRT in HF patients are patient selection, general heart failure treatment and CRT delivery characteristics [9].

Within CRT delivery characteristics, LV lead placement (LVLP) appears to be one of the main contributors to the extent of benefit derived from CRT [10,11]. Current guidelines suggest that the LV lead is to be placed fluoroscopically in the non-apical posterolateral region [6]. Many studies, however, have focused on more distinguished ways to determine the optimal LV lead location. Highly developed cardiac imaging techniques such as echocardiography, electrocardiographic imaging (ECGi), CMR, etc., have been used to establish myocardial properties, including myocardial scar, and the latest electrically or mechanically activated segments to determine the best location for the LV lead to be placed [10,11,12,13,14,15]. Unfortunately, some of the previously mentioned imaging modalities are expensive or not widely available and do not offer the implanter direct feedback on the extent of resynchronization achieved at every evaluated LVLP. Tools that offer the possibility of direct feedback during implantation, such as noninvasive epicardial electrical mapping (ECGi) or the ECG belt, are quite cumbersome to use due to the numerous electrodes and have not proven to provide significant improvements with respect to outcomes [16,17]. Hence, the 12-lead ECG-derived parameter QRS area should be (re)considered to provide the additional guidance needed to optimize LVLP in CRT patients.

## 2. Twelve-Lead ECG-Derived Markers of Dyssynchrony; QRS Duration, Morphology and the Potential Role of Vectorcardiographic QRS Area

Currently, invasive cardiac mapping is considered the golden standard for the evaluation of the heart’s electrical conduction system and its disturbances. Nonetheless, the practical execution of this type of mapping proves to be challenging and demands the utilization of an ECG within the healthcare domain. According to the ESC guidelines, the suitability of patients for CRT is accessed through QRS morphology and QRS duration. Limitations in the use of these ECG-derived parameters have been acknowledged before [7,18,19]. While QRS duration involves no interpretation for assessment, it contains no information regarding the direction of electrical activation. Regarding the latter aspect, the QRS morphology can provide knowledge of the direction of the electrical impulse. Nevertheless, the interpretation of QRS morphology relies heavily on the observer’s perception and is therefore accompanied by large inter-observer variability [20]. Whereas assessment of QRS duration and QRS morphology is recommended by the ESC guidelines in patient selection for CRT [6], there has not been any consistent, nor convincing evidence for the usage of these parameters neither in LVLP nor in optimization of resynchronization thereafter.

The role of the 12-lead ECG in assessing the contribution of LVLP in CRT has been evaluated by several observational studies. Brandtvig et al. showed that lateral LV lead placement was associated with a larger reduction in QRS duration (−13 ± 27 ms vs. −3 ± 24 ms, *p* < 0.001) compared to anterior, apical and inferior pacing [21]. Borgquist et al. investigated the correlation between the reduction in QRS duration and clinical outcome in a study encompassing 257 CRT patients with an LBBB QRS morphology at baseline. Results indicate that QRS duration reduction of more than 14ms (median value) was associated with a lower risk of death or HF hospitalization (0.60; 0.38–0.96, *p* = 0.03) [22]. These results were also validated by Molhoek et al. in a prospective study involving 61 patients, using a composite CRT clinical response score of improved NYHA Class, 6 min walking distance and quality of life score. The responder group showed significantly more shortening in QRS duration (179 ± 30 ms to 159 ± 25 ms, *p* < 0.01 vs. 171 ± 32 ms to 160 ± 26 ms) at 6 months [18]. Furthermore, this study investigated the sensitivity and specificity of the reduction in QRS duration as a predictor of benefit from CRT; however, the results were not clear enough to define a clear cut-off value for the extent of QRS duration reduction that predicts response. Recent meta-analyses including observational studies with both echocardiographic and clinical outcomes confirmed the aforementioned results, however, the analysis concluded that large prospective studies are lacking to confirm the association [23]. These associations regarding the shortening of QRS duration may potentially offer better differentiation within subgroups of the CRT patient population in terms of predicting the CRT benefit, in contrast to the assumed causal association. Unlike paced QRS duration (and hence reduction), paced QRS morphology has scarcely been evaluated as a marker of response to CRT. Bode et al. retrospectively analyzed 68 CRT patients and investigated the biventricular paced QRS morphology in association with echocardiographic response (LVEF increase ≥ 7.5%). They found that ECG evidence of biventricular capture (a positive vector in V1 and/or negative vector in lead I) was associated with a significantly higher chance of response (69% vs. 11%, sensitivity 94%, specificity 53%, *p* < 0.0001) [24]. In summary, paced reduction in QRS duration and paced QRS morphology have shown promising associations with CRT response. However, causal associations cannot be made as studies on the guidance of LVLP are lacking. Moreover, proven limitations in the use of these markers in patient selection for CRT may similarly apply to their use in guiding LVLP.

**Figure 1:**
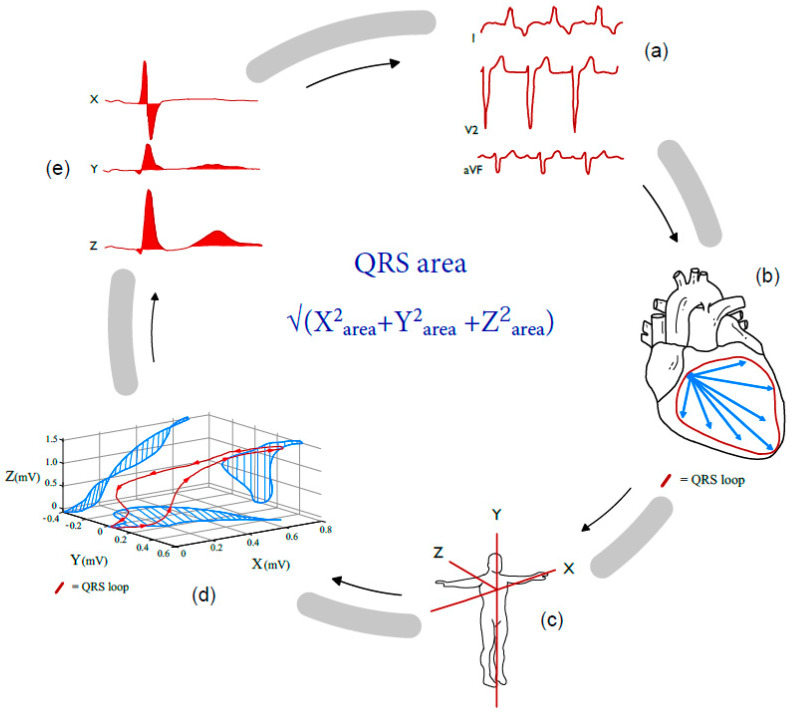
Reconstruction of QRS area. (**a**) Extracting a 12-lead ECG from patient. (**b**) Vectors resultants of the QRS complex over time creating a QRS loop. (**c**) QRS loop in a 3D perspective. (**d**) Reflecting the QRS loop areas in X-, Y- and Z-plane. (**e**) Calculating the QRS area by sum of the X-, Y-, and Z-area.

Recently, vectorcardiographic QRS area has been introduced as a promising tool for the optimization of LVLP and pacing configuration. Using vector-based electrical information derived from the 12-lead ECG, the QRS area can provide information on ventricular resynchronization achieved at any LV lead location, as well as the selected electrode(s) on the LV lead. Vectorcardiographic (VCG) QRS area is a biomarker that can directly be reconstructed from the 12-lead ECG with the help of the Kors conversion matrix. The results of a reconstructed VCG resemble the true direct measured vectorcardiogram in patients [25]. The technique uses the information contained in the 12-lead ECG to create a 3D vector loop of electrical activation along three orthogonal directions (X, Y and Z) that encompasses all the electrical information of the heart (both depolarization and repolarization) (see Figure 1).

Within the context of VCG parameters, the QRS area holds particular significance for CRT, since it encompasses two features: amplitude and duration of the ventricular depolarization. This parameter refers to the total area encompassed by the QRS complex on the vectorcardiogram and serves as an indicator for the sum of unopposed electrical forces activating the myocardial ventricles. Due to the basic anatomical differences between the right and left ventricles, the QRS area also encompasses information about the direction of activation. As the LV mass is significantly larger than that of the RV, (delayed) ventricular activation directed toward the LV will have a far higher amplitude than ventricular activation directed toward the RV. Since the QRS area incorporates the QRS duration and amplitude, it distinguishes itself from other ECG parameters and thereby provides more accurate and objective information regarding the electrical activation of the ventricles than QRS duration and morphology. Unlike QRS morphology, its assessment does not depend on the observer’s interpretation, as it includes quantitative information on both duration and amplitude of the ventricular activation, irrespective of the direction or manner of ventricular activation (i.e., native conduction or stimulation by means of cardiac pacing). The latter implies its usefulness in both selection of potential candidates for CRT, as well as patients treated with CRT. Studies have shown that baseline QRS area provides better differentiation in mortality risk compared to QRS duration and QRS morphology (AUC, 0.61 vs. 0.51 and 0.55, respectively; *p* < 0.001), demonstrating its usefulness in patient selection [7,19]. QRS area furthermore demonstrated superior efficacy in discerning patients exhibiting echocardiographic remodeling in response to CRT compared to both QRS morphology and duration (AUC, 0.69 versus 0.58 and 0.58, respectively; *p* < 0.001) [7]. The results of these studies show that the QRS area is more precise than QRS duration and morphology in differentiating between patients who improve from CRT compared to those who do not improve from CRT, based on the baseline 12-lead ECG depicted LV dyssynchrony [7,26,27,28,29]. Considering the fact that within CRT not only patient selection influences the CRT outcome, but also the location of the LV lead, it is worthwhile to thoroughly examine the role of QRS area within LVLP.

## 3. The Importance of LV Lead Position in Benefit from CRT

The importance of the location of the LV lead in the effect of CRT first became clear from subanalyses of the initial landmark trials in CRT [30,31]. A subanalysis of the Multicenter Automatic Defibrillator Implantation Trial-Cardiac Resynchronization Therapy (MADIT-CRT) trial, a prospective study with 799 participants, assessed final LVLP on coronary venograms and chest x-rays. The investigation conducted a comparative analysis of the impact of the following three LV lead locations: basal, midventricular and apical region on both mortality rates and the incidence of heart failure. The outcome implicated that basal and mid-ventricular lead placement were superior to apical lead implantation (mostly mid-cardiac veins) (hazard ration (HZ) = 1.72; 95% CI, 1.09 to 2.71; *p* = 0.019) [10]. Another subanalysis of the MADIT-CRT trial by Kutyifa et al. indicated that an LV lead placed in an anterior position, compared to a lateral or posterior position, corresponded with a significantly increased risk of death or ventricular arrhythmias [32]. These outcomes have led to the current recommendation for LV lead placement in a non-apical posterolateral region according to the ESC guidelines from 2021.

After the aforementioned subanalyses of the landmark trials, attention shifted to other elements that influence the effect of LVLP on the clinical outcome of CRT. Studies have revealed that scar tissue intervenes with the electrical signaling of the heart. Activation in the infarcted region is characterized by local delays and fractionated, low-amplitude extracellular electrograms [9,10]. Ypenburg et al. demonstrated that the extent of scar tissue is negatively associated with the response to CRT. Patients who did not improve in both clinical and echocardiographic parameters after CRT had less viable myocardial tissue (11–13 segments versus 5–10 segments) (*p* < 0.001) [33]. Bose et al. elaborated on this subject in a study incorporating 160 patients with ischemic cardiomyopathy, where the role of scar tissue in relation to the final LV lead position was assessed. A Cox proportional hazard model revealed that LV leads placed in areas with scar or scar in combination with ischemia independently are associated with more HF hospitalizations and increased mortality rates [11]. Based on the former observations, the extent of myocardial scarring has since been established to negatively influence the general outcome of CRT [14,34,35,36], especially when the final LVLP rests in an area of scarring.

**Figure 2:**
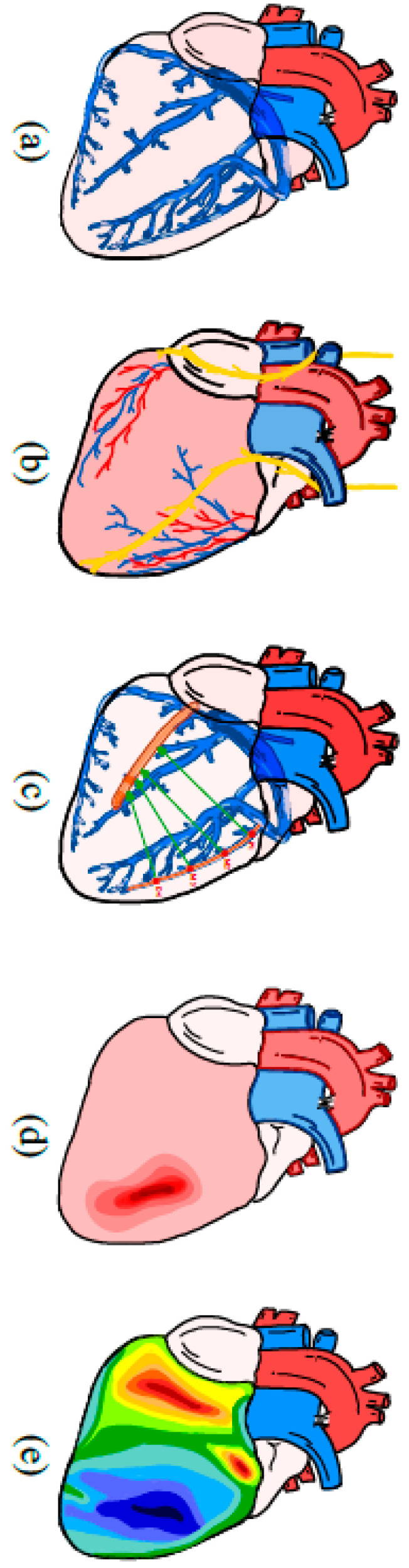
Factors playing a role in left ventricular lead placement. (**a**) Variations in coronary venous anatomy. (**b**) Proximity of the phrenic nerve. (**c**) Pacing characterisitcs (threshold). (**d**) Proximity of myocardial scar. (**e**) Site of latest left ventricular electrical activation.

However, tissue characteristics such as fibrosis and scarring are not the only factors that influence the effectiveness of LVLP in the context of CRT (see Figure 2). Theoretically, LVLP at the site of the latest activation should result in capturing the largest dyssynchronic myocardial area, and hence has the greatest potential for resynchronization. Taylor et al. studied the role of LV leads placed in areas with or without myocardial scarring, and the site of the latest (mechanical) activation (concordant LV lead) in correlation to LV reverse remodeling and clinical outcomes post-implantation (primary outcome was death or HF hospitalization) [14]. Data demonstrated that concordant LV leads compared to non-concordant LV leads were associated with greater LV reverse remodeling and improved clinical outcomes (adjusted odds ratio (aOR) 0.26, 95% CI 0.12–0.58; aOR 0.24; 95% CI 0.12–0.49, respectively for LMA and scar burden) [14]. Conformingly, the Targeted Left Ventricular Lead Placement to Guide Cardiac Resynchronization Therapy (TARGET) trial indicated that speckle-tracking echocardiography guided LVLP, directing the LV lead to the latest mechanically activated region, compared to the standard (anatomical) LVLP was superior (clinical response rate defined as ≥1 improvement in New York Heart Association functional class) in terms of all-cause mortality, combined all-cause mortality, and heart failure-related hospitalization (83% vs. 65%, *p* = 0.003) [15]. In order to place the LV lead in the latest activated region of the LV, various techniques can be employed. In a randomized controlled trial with 122 participants, Stephansen et al. compared two techniques: (1) electrically guided LVLP (the LV lead was employed for systematic electrical mapping by measuring QLV in basal, mid, and apical segments of CS branches) and (2) imaging-guided strategy regarding the influence on LVEF through computed tomography (CT) venography and speckle-tracking echocardiography. The results show that electrically guided CRT performed equally compared to the imaging-guided approach in improving LVEF (*p* = 0.09) [37]. Another study carried out by Nguyên et al. combined coronary venous electroanatomic mapping (EAM) with delayed enhancement cardiac magnetic resonance imaging (DE-CMR) in eighteen patients with focal scarring. Through the combination of these two techniques, the researchers were able to place the LV lead in ten out of eighteen patients away from the scar and near the latest electrically activated area. Results show a feeble but significant correlation between voltages and DE-CMR scar fraction (partial correlation R − 0.161, *p* = 0.001), indicating a useful implementation of these two techniques within optimizing LVLP [38].

Other tools for visualizing the heart’s electrical conduction have been proposed such as noninvasive epicardial electrical mapping (ECGi), recording detailed electrical activation maps in relation to cardiac anatomy as captured by cardiac CT scanning and, more recently, the ECG belt device. However, none of the aforementioned highly distinguished determinations of the site of latest electrical or mechanical activation have unequivocally shown to be equal compared to standard anatomical LVLP, as suggested by current guidelines [17]. All these techniques require expert knowledge and entail significant costs. Moreover, the techniques propose a target for the LVLP before or during implantation but do not allow evaluation of the chosen LVLP at implantation and subsequent replacement at apparent insufficient resynchronization at the chosen LVLP. This asks for easily available methods that allow real-time guidance of LVLP and evaluation of chosen LVLP during implantation for the optimal benefit from CRT. The traditional 12-lead ECG, readily available at any CRT clinic and providing real-time feedback on any LV lead position evaluated during implantation, seems the most appropriate candidate for this purpose.

## 4. QRS Area in LVLP

In the context of LVLP and associated timing optimization of biventricular pacing, QRS area theoretically entails information about the amount of (LV) mass captured within its LV-RV fusion pacing activation front, and the reduction in QRS area. The QRS area can be compared to the baseline and may therefore be used to guide LVLP as well as optimization of pacing settings thereafter. A reduced QRS area provides insight into the extent of the total ventricular activation time as well as the amplitude of unopposed forces. Studies have shown that baseline QRS area is correlated with the amount of myocardial tissue (mass) [39] as well as with the presence of myocardial scar (loss of electrically viable myocardial tissue) [40]. The correlation between myocardial scarring and QRS area was investigated by Nguyên and colleagues. In 33 patients qualified for CRT, VCG metrics were obtained from ECG-synthesized VCGs, and myocardial properties were collected using CMR. Response to CRT was determined as ≥15% reduction in LV end-systolic volume after six months of follow-up. QRS area was inversely correlated with focal scarring (R = −0.44–−0.58 for scar, *p* ≤ 0.010) and it confirmed the association of baseline QRS area to predict CRT response with an AUC of 0.737 (*p* = 0.022) [40]. In 26 patients with an optimally deployed quadripolar LV lead who underwent assessment of LV-pressure (ΔLV dP/dt_max_) during CRT using LVLPs, Okafor et al. investigated the correlation between QRS area, myocardial scar and LVLP. Results showed that QRS area and the extent of myocardial scarring are correlated (r = 0.35, *p* = 0.003) [41]. Moreover, LV leads placed in a scarred LV segment were associated with an increased QRS area (+22.2 ± 58.4 μVs, *p* < 0.001) in comparison to LV leads placed away from the scar or with no scarring at all (−3.28 ± 38.1 μVs and −43.8 ± 36.8 μVs *p* < 0.001).

The potential role of QRS area to optimize LVLP during an implantation procedure and optimization of pacing settings thereafter was further investigated by Ghossein et al. in a retrospective analysis encompassing 52 CRT patients. The objective of this study was to examine whether a decrease in QRS area (∆QRS area) was linked to enhancements in acute LV hemodynamics through pacing at various LV sites and whether the reduction in QRS area could serve as a guide for optimal placement of the LV lead. Lastly, they compared ∆QRS area with ∆QRSd in correlation with the acute hemodynamics. Acute hemodynamic response (AHR) was assessed by measuring differences in LV pressure build-up (∆LVdP/dt_max_). The study evaluated a total of 188 different pacing sites. The analyses revealed a strong correlation between AHR and QRS area (median R = 0.76, IQR 0.35; 0.89). The same correlation could not be found for ∆QRSd. Within the scope of selecting the right electrodes, the results showed that the pacing of the proximal electrode was accompanied by an increased AHR (*p* = 0.004) and ∆QRS area (*p* = 0.003). Again, this correlation did not apply for QRSd (*p* = 0.77) [42]. These results were confirmed in the aforementioned study by Okafor et al. Also using AHR as a surrogate outcome, they found that QRS area reduction (in contrast to baseline QRS area) was significantly associated with ∆LVdP/dt_max_ (r = −0.68; *p* < 0.001). Moreover, QRS area reduction correlated with AHR at different LV lead positions (73.1% of patients showed the largest QRS area reduction at the site of optimal ∆LVdP/dt_max_). This way, they estimated that 15% of patients could be ‘guided’ from hemodynamic non-response to hemodynamic response, comparing worst to best LV lead locations [41]. The sensitivity of QRS area reduction to optimize pace settings after the implantation phase was further evaluated by Engels et al. In a study of 120 patients implanted with a CRT device, VCG analyses were conducted with pacing at nominal atrioventricular delays (BiV nominal) and Biv with SyncAV programming (Biv + SyncAV). Results showed QRS area to be sensitive to the SyncAV programming, resulting in a stronger decrease in QRS area in patients with Biv + SyncAV (53 ± 30 mV ∗ ms, *p* = 0.06 vs. BiV Nominal) [43]. In a subsequent study, this group evaluated the feasibility of the use of intracardiac electrogram (EGM)-based vector loops to optimize pacing (LV fusion pacing with intrinsic conduction). Using AHR ((∆LVdP/dt_max_) as a marker of response, they used an LV pacing protocol adjusting AV pace delays trying to find the optimal fusion with intrinsic conduction and its relation with ECG-based vectorloops. They found that by using these EGM-based vector loop signals, they were able to find pacing settings with the largest AHR benefit. Furthermore, they found that the EGM-derived vector-loop-based optimization was able to consistently result in improved AHR as compared to known optimization algorithms [44].

## 5. QRS Area in Conduction System Pacing

As conduction system pacing, in particular left bundle branch area pacing (LBBAP), is currently making its way as an alternative to biventricular pacing in CRT, QRS area may also have a role in guiding LBBA pacing lead location, or in other words, assessing quality of conduction system pacing. Securing LBB capture seems essential for LBBAP to derive similar success as biventricular pacing in CRT. Liu et al. investigated the effect of stimulating different locations of the left bundle branch (LAFP, left anterior fascicular pacing; LBBP, left bundle branch pacing; LBTP, left bundle trunk pacing; LPFP, left posterior fascicular pacing) on the ∆QRS area in 91 patients implanted with an LBBAP system for a conventional brady pacing indication. The study showed a difference in QRS area when LPFP was compared to LBBP. LBBP showed no significant difference in QRS area after stimulation (35.1 μVs) compared to intrinsic conduction (34.7 μVs, (*p* = 0.98)), whereas LPFP showed a significant increase in QRS area from 35.7 μVs to 43.4 μVs, (*p* = 0.01), compared to intrinsic conduction [45]. These data are supported by a recent study by Heckman et al. encompassing 50 patients implanted with an LBBAP device indicated for bradycardia. Paced ECGs at RV septal pacing (RVSP), LV septal pacing (LVSP) and left bundle branch pacing (LBB) were evaluated. The results show that QRS area decreased from 82 ± 29 µVs during RVSP to 46 ± 12 µVs with LV septal pacing (LVSP) and 38 ± 15 µVs with LBB pacing [46].

These studies suggest that the procedural QRS area may provide insight into the exact LBBA pacing location in patients implanted with an LBBAP system for CRT, ensuring optimal therapy. Whereas currently, many sites incorporating LBBAP as a strategy for CRT still rightfully use advanced electrophysiological (EP) measurements to ensure conduction system capture, the apparent success of LBBAP is catalyzing widespread adaptation of this technique. Non-EP centers will therefore need alternative measures to ensure conduction system capture in LBBAP. QRS area may therefore play an important role in the near future, ensuring high-quality procedures at a minimal effort. Even though QRS area is not currently widely available, theoretically, it could easily be incorporated into standard ECG equipment for automated calculation, similar to QRS duration.

## 6. Conclusions and Future Perspectives

Over recent decades, QRS area has been shown to be a very promising marker. Even though research has mainly focused on its role in patient selection for CRT, QRS area may theoretically be a very interesting marker to guide LVLP. Few studies have shown its potential as a marker for the extent of resynchronization in both LVLP for conventional biventricular pacing, as well as CRT by means of conduction system pacing. Whereas results are promising, prospective studies on the association of QRS area-guided LVLP and long-term outcomes are lacking. Moreover, the strength of evidence for the use of QRS area in the upcoming field of LBBAP for CRT needs to be extended for both short- and long-term outcomes.

## Data Availability

Not applicable.
